# Lower cardiotoxicity of CPX-351 relative to daunorubicin plus cytarabine free-drug combination in hiPSC-derived cardiomyocytes in vitro

**DOI:** 10.1038/s41598-023-47293-4

**Published:** 2023-11-29

**Authors:** Marie C. Fortin, Andrew S. LaCroix, Tom N. Grammatopoulos, Lei Tan, Qi Wang, Dino Manca

**Affiliations:** 1 Jazz Pharmaceuticals, 2005 Market Street, 21St Floor, Philadelphia, PA 19103 USA; 2 StemoniX, Maple Grove, MN USA; 3 BioEnergetics LLC, Boston, MA USA

**Keywords:** Drug safety, Toxicology, Cancer, Stem cells, Cardiology, Oncology

## Abstract

Liposomal formulations are hypothesized to alleviate anthracycline cardiotoxicity, although this has only been documented clinically for doxorubicin. We developed an in vitro multiparametric model using human-induced pluripotent stem cell-derived cardiomyocytes (hiPSC-CM) to assess the relative toxicity of anthracyclines across formulations. Proof of concept was established by treating hiPSC-CM with equivalent concentrations of free and liposomal doxorubicin. The study was then repeated with free daunorubicin plus cytarabine and CPX-351, a dual-drug liposomal encapsulation of daunorubicin/cytarabine. hiPSC-CM were treated with free-drug or liposomal formulations for 24 h on Days 1, 3, and 5 at equivalent concentrations ranging from 0 to 1000 ng/mL and assessed on subsequent days. Free-drug treatment resulted in concentration-dependent cumulative cytotoxicity (microscopy), more profound decrease in ATP levels, and significant time- and concentration-dependent decreases in oxygen consumption versus liposomal formulations (p < 0.01). Repeated free-drug exposure also resulted in greater release of biomarkers (cardiac troponin I, FABP3) and lactate dehydrogenase, as well as in a biphasic rhythmicity response (initial increase followed by slowing/quiescence of beating) indicating significant injury, which was not observed after repeated exposure to liposomal formulations. Overall, liposomal formulations were considerably less toxic to hiPSC-CM than their free-drug counterparts. Clinical data will be needed to confirm findings for CPX-351.

## Introduction

Anthracyclines, such as doxorubicin and daunorubicin, which are both included on the World Health Organization’s list of essential medicines^1^, have been a pillar of cancer therapy since the 1970s. They are among the most prescribed chemotherapeutic agents and are used for the treatment of leukemias, lymphomas, and breast, stomach, uterine, ovarian, bladder, and lung cancers^2–4^. Despite the proven benefits of anthracyclines, their clinical utility is limited by their cardiotoxicity, which can present as an immediate pericarditis/myocarditis syndrome, early-onset progressive chronic heart failure developing during or shortly after therapy, or late-onset cardiotoxicity occurring sometimes years after chemotherapy. Since the heart has a limited regenerative capacity, repeated insults or injuries ultimately overwhelm the organ’s capacity to produce an adaptive response^5^. This can lead to maladaptive responses, such as inflammatory cell infiltration, fibrosis, and cell death, which ultimately present clinically as cardiomyopathy or clinical heart failure^6^. The cumulative dose of anthracyclines has been shown to be the main determinant for the development of cardiotoxicity in patients receiving anthracycline-based treatment. Potential risk factors, such as combination therapy, radiotherapy, older age, pre-existing conditions, tobacco use, and previous exposure to cardiotoxic agents, are also important contributors to cardiotoxicity^7^.

Although oxidative stress is considered a key mechanism for anthracycline cardiotoxicity, there are many potential other mechanisms, such as DNA damage, suppression/dysregulation of genes, dysregulation of autophagy, endoplasmic reticulum stress, and—importantly—mitochondrial dysfunction^8,9^. It was hypothesized by Wallace et al. that doxorubicin treatment results in oxidation of mitochondrial DNA (mtDNA), inhibiting its ability for replication and expression, causing longer-term damaging effects and cardiotoxic memory^10^. The prolonged impaired expression of critical oxidative phosphorylation subunits affects mtDNA copy number, decreases mitochondrial adenosine triphosphate (ATP) production, and increases production of reactive oxygen species, which could eventually develop into cardiomyopathy.

The incidence of cardiotoxicity has been shown to increase dramatically as the cumulative anthracycline dose increases. For example, the probability of developing cardiomyopathy is estimated to be 1% to 2% at a cumulative lifetime dose of 300 mg/m^2^ of doxorubicin hydrochloride, 5% at a dose of 400 mg/m^2^, 5% to 8% at a dose of 450 mg/m^2^, and 6% to 20% at a dose of 500 mg/m^2^^3^. Similarly, cumulative doses of daunorubicin > 550 mg/m^2^ have been associated with an increased incidence of drug-induced congestive heart failure^2^. Liposomal formulations may help alleviate some of the cardiotoxic effects of anthracyclines by altering the pharmacokinetics (PK), free-drug concentrations, and—ultimately—myocardium tissue exposure^11^. Smith et al. assessed the risk of early and late cardiotoxicity of different anthracyclines in the treatment of malignancies^12^. Their study demonstrated that the risk of clinical and subclinical cardiotoxicity was considerably lower with liposomal versus non-liposomal doxorubicin (odds ratio = 0.18 [95% confidence interval: 0.08, 0.38]). There was also a correlation between the maximum concentration (C_max_) and the development of some adverse cardiovascular effects^12^.

Intensive chemotherapy with the combination of daunorubicin plus cytarabine has been a standard-of-care treatment for acute myeloid leukemia (AML). To improve outcomes and limit systemic toxicity (including cardiotoxicity), CPX-351 (United States: Vyxeos; European Union/United Kingdom: Vyxeos liposomal) was developed as a liposomal encapsulation of daunorubicin and cytarabine in a synergistic 1:5 molar ratio. It is approved for the treatment of newly diagnosed, therapy-related AML or AML with myelodysplasia-related changes in adults and children aged ≥ 1 year in the United States and adults aged ≥ 18 years in Canada, the European Union, and the United Kingdom^13,14^. In a preclinical study in which rats were treated with CPX-351 or the free-drug combination of daunorubicin plus cytarabine at equivalent doses, the C_max_ for daunorubicin was lower following the administration of CPX-351 compared to the free drug^15^. The heart-to-plasma concentration ratio was also approximately 100-fold lower for daunorubicin following the administration of CPX-351 compared to the free drug.

In our study, we utilized human-induced pluripotent stem cell–derived cardiomyocytes (hiPSC-CM) to characterize and compare the relative cardiotoxicity of CPX-351 versus free daunorubicin plus cytarabine, given that intrinsic biological differences between species result in challenges in studying cardiotoxicity in preclinical models because animals often succumb from gastrointestinal or bone marrow toxicity before any signs of cardiotoxicity are observed. hiPSC-CM were first reported in 2009, and since then the field of cardiotoxicity safety assessment has been revolutionized by hiPSC-CM, which are recognized and accepted by regulatory agencies (Comprehensive In Vitro Pro Arrhythmia Assay [CiPA], US Food and Drug Administration, and Health Canada) for safety evaluation^16–19^. hiPSC-CM are obtained from human cells (typically fibroblasts or peripheral blood mononuclear cells), which are induced into a pluripotent state and then re-differentiated into CM that exhibit spontaneous rhythmicity. Whole-genome transcript profiling and immunohistochemical characterization demonstrated that the iCell CM used in this study show a stable human cardiac gene expression profile with protein expression and localization adequate for proper cardiac function, including tight junctions, actinin, and ion channel expression^20^.

To evaluate the external validity of this in vitro model, we first sought to determine whether it could recapitulate what had been documented for liposomal versus free doxorubicin in the clinic (i.e., higher cumulative doses of the liposomal formulation can be administered before any cardiotoxicity is observed). The dosing paradigm was designed to emulate the clinical regimen of CPX-351. The concentrations chosen were also relevant, encompassing the C_max_ values obtained with clinical administration of free daunorubicin or doxorubicin (0–1000 ng/mL)^21^. For reference, in vitro median half-maximal efficacious concentrations (EC50s) of doxorubicin and daunorubicin to sensitive cancer cells are usually < 10 ng/mL^22,23^. The repeated-administration design was intended to evaluate the cumulative toxicity, and as such, higher concentrations could not be used because they would be lethal to the cells after a single exposure^24^. In terms of endpoints, we took into consideration the multiplicity of mechanisms by which anthracyclines can cause toxicity and therefore included cellular imaging via microscopy as well as assessments of viability, function, mitochondrial function, and biomarkers related to cardiomyocyte damage. We then employed the same design and conditions to compare the toxicity of CPX-351 to equivalent doses of a free-drug combination of daunorubicin plus cytarabine to evaluate whether the liposomal formulation of CPX-351 minimizes cardiotoxicity.

## Results

### hiPSC-CM viability is preserved following treatment with liposomal anthracyclines

#### Bright-field microscopy

**Figure 1:**
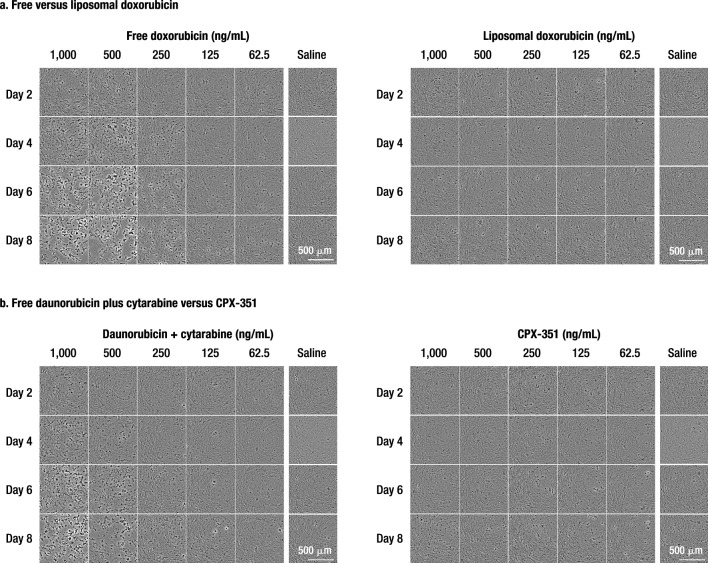
Cell morphology following treatment with free drugs versus liposomal formulations. (**a**) Free versus liposomal doxorubicin. (**b**) Free daunorubicin + cytarabine versus CPX-351. Decreased viability is indicated by cell peeling and increased contrast from background and is evident as early as Day 4 for the highest concentrations of free drugs.

On Day 1, prior to dosing, cells were confluent and beating synchronously. On Day 2, there was little difference between the treatment groups, with the exception of free doxorubicin, which showed evidence of damage starting at concentrations of 500 ng/mL. On Day 4, cells treated with ≥ 500 ng/mL of free drugs displayed morphological changes and were visibly not as healthy as those treated with lower concentrations, liposomal formulations, or treated only once. On Day 6, both doxorubicin and daunorubicin + cytarabine treatments induced cell death starting at 500 ng/mL with more pronounced effects at 1000 ng/mL. Additionally, doxorubicin-treated cells at 250 ng/mL had already started displaying signs of damage. By Day 8, cells treated with free drugs were visibly fewer in number and did not appear viable (spherical shape and detached) at concentrations ≥ 250 ng/mL for doxorubicin and daunorubicin (+ cytarabine), although not as pronounced for the latter. Such effects were not evident with cells treated with liposomal doxorubicin or CPX-351 through Day 8. Figure 1 provides representative images of the hiPSC-CM treated with a range of concentrations of liposomal and free anthracyclines for one 24-h treatment period (Day 2), two 24-h treatment periods (Day 4), three 24-treatment periods (Day 6), or three 24-h treatment periods plus a 48-h off-treatment period (Day 8).

#### ATP content and lactate dehydrogenase (LDH) release

**Figure 2:**
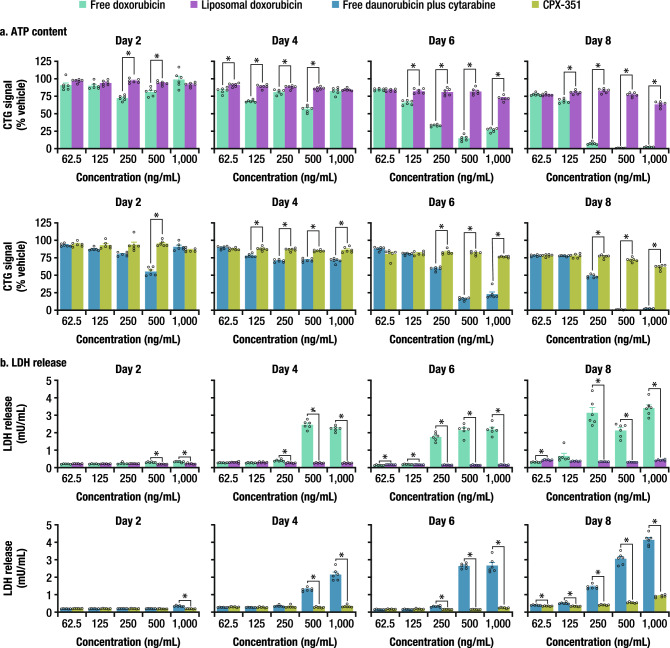
ATP content and LDH release following treatment with free drugs versus liposomal formulations. (**a**) ATP content. (**b**) LDH release. *ATP* adenosine triphosphate, *CTG* CellTiter-Glo, *LDH* lactate dehydrogenase, *SEM* standard error of the mean. Data are mean ± SEM. The number of replicates was six for each assay. *Denotes q < 0.01, free versus liposomal.

Cell viability was assessed using two complementary methods (Fig. 2; Suppl. Table 2). On Day 2, following a single 24-h treatment, there were statistically significant, but probably not biologically meaningful, differences in normalized ATP content between some of the treatment groups, as the statistically significant differences did not follow a concentration–response. On Day 4, although some differences between the cells treated with liposomal formulations versus their respective free drug conditions were observed, again these changes did not follow a concentration response and could have been due to assay variability. By Day 6, the ATP content was consistently and statistically significantly higher in cells that were treated for three 24-h periods with the liposomal formulations compared to those treated with free drugs doxorubicin or daunorubicin (+ cytarabine) at concentrations ≥ 250 ng/mL. This difference between liposomal and free-drug formulations was more profound on Day 8 despite not having received any additional treatment since Day 5. Conversely, LDH release was increased by repeated exposure to the free drugs in a comparable manner, although biologically meaningful differences in membrane integrity were detected as early as Day 4, after only two treatments. A concentration- and cumulative exposure–dependent response was observed, with increasing amounts of LDH released in the media detected with increasing concentrations and repeated exposures. There were no appreciable changes in LDH release with increasing concentrations of either liposomal formulation, even on Day 8, suggesting liposomal formulations are largely effective at protecting hiPSC-CM from anthracycline toxicity. Overall, LDH seemed slightly more sensitive than ATP as a biomarker of viability.

### hiPSC-CM function is preserved following treatment with liposomal anthracyclines

**Figure 3:**
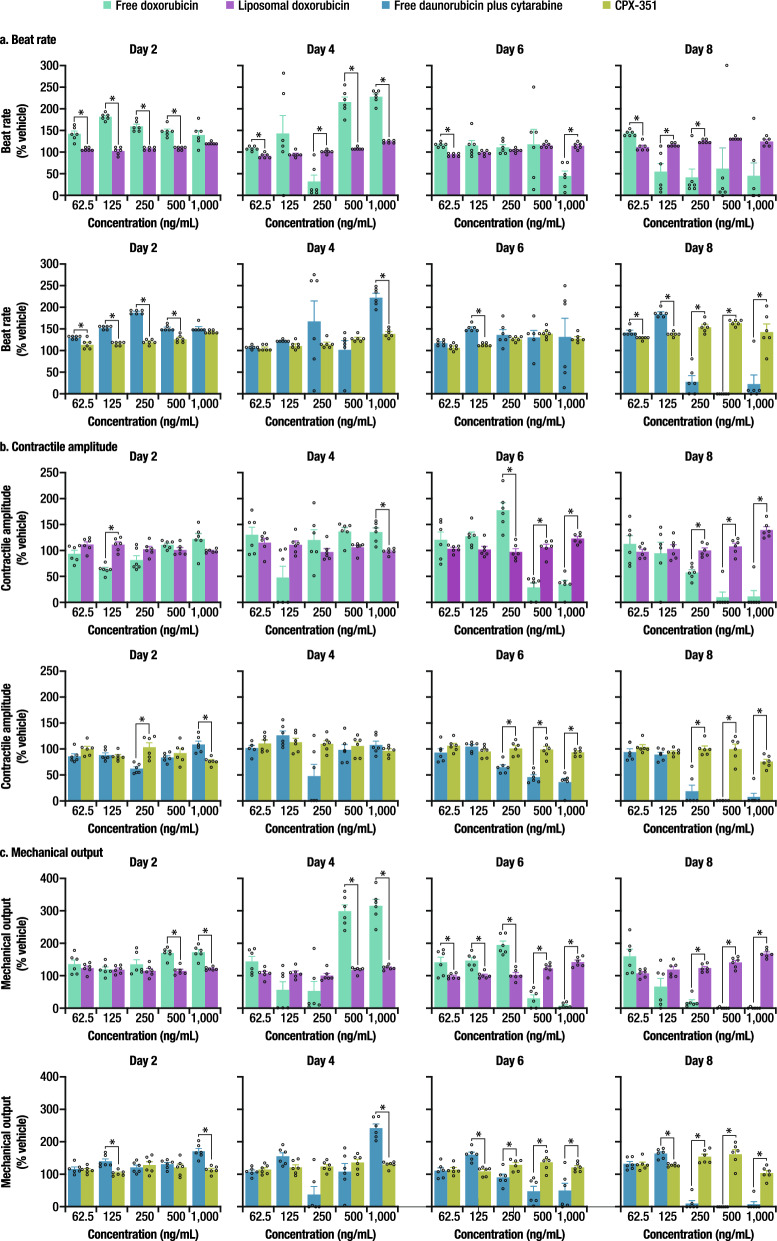
(**a**) Beat rate, (**b**) contractile amplitude, and (**c**) mechanical output following treatment with free drugs versus liposomal formulations. *SEM* standard error of the mean. Data are mean ± SEM. The number of replicates was six for each assay. *Denotes q < 0.01, free versus liposomal.

Image analysis was used to evaluate the effect of liposomal and free anthracycline exposures on hiPSC-CM function. Three specific measures of clinical relevance were evaluated (Fig. 3): the beat rate, which is analogous to the heart rate; the contractile amplitude, which would correlate to the ejection fraction; and the mechanical output (beat rate × contractile amplitude), which would conceptually be equivalent to the cardiac output (heart rate × ejection fraction)^25^. The hiPSC-CM beat rate response to the free drug exposure appeared biphasic. Following the first 24-h treatment, cells exposed to free doxorubicin or daunorubicin (+ cytarabine) generally displayed higher beat rates than cells treated with the liposomal formulations. On Day 4, after two 24-h treatments, cells exposed to free doxorubicin at concentration ≥ 500 ng/mL or daunorubicin at 1000 ng/mL (+ cytarabine) showed a faster beat rate compared to those treated with the liposomal formulations. By Day 6, cells treated with 1000 ng/mL free doxorubicin beat more slowly. On Day 8, despite no additional exposure, the cells treated with concentrations ≥ 125 ng/mL free doxorubicin or concentrations ≥ 250 ng/mL free daunorubicin also slowed their beating. The cells treated with the liposomal formulations showed a modest increase in beat rate compared to the controls on Day 8 and continued to beat throughout the study. Similar results were seen in absolute beat rate values (Suppl. Table 3). On Days 6 and 8, contractile amplitude was significantly lower with free doxorubicin at concentrations ≥ 500 ng/mL and free daunorubicin (+ cytarabine) at concentrations ≥ 250 ng/mL compared with the liposomal formulations. The mechanical output showed a biphasic response, consistent with changes in beat rate. On Days 2 and 4, the mechanical output was greater with free doxorubicin at concentrations ≥ 500 ng/mL and free daunorubicin (+ cytarabine) at 1000 ng/mL. By Day 6, the mechanical output was lower with both free drugs at concentrations ≥ 500 ng/mL compared to the liposomal formulations, but generally higher at lower concentrations of the free drug, consistent with a time and concentration-dependent response. On Day 8, the mechanical output remained significantly lower with both free drugs at concentrations ≥ 250 ng/mL. The modest increase in beat rate with liposomal formulations on Day 8 may appear to be the initial phase of the response of CM to anthracycline exposure as seen by Day 2 with the free drugs. It is hypothesized that this increase was an aftereffect of the slow leak of anthracyclines out of the liposomes or of liposome breakage during media changes and resulting mild repeated exposure over the treatment period.

### Cardiac biomarker release profile diverges following treatment with liposomal versus free anthracyclines

**Figure 4:**
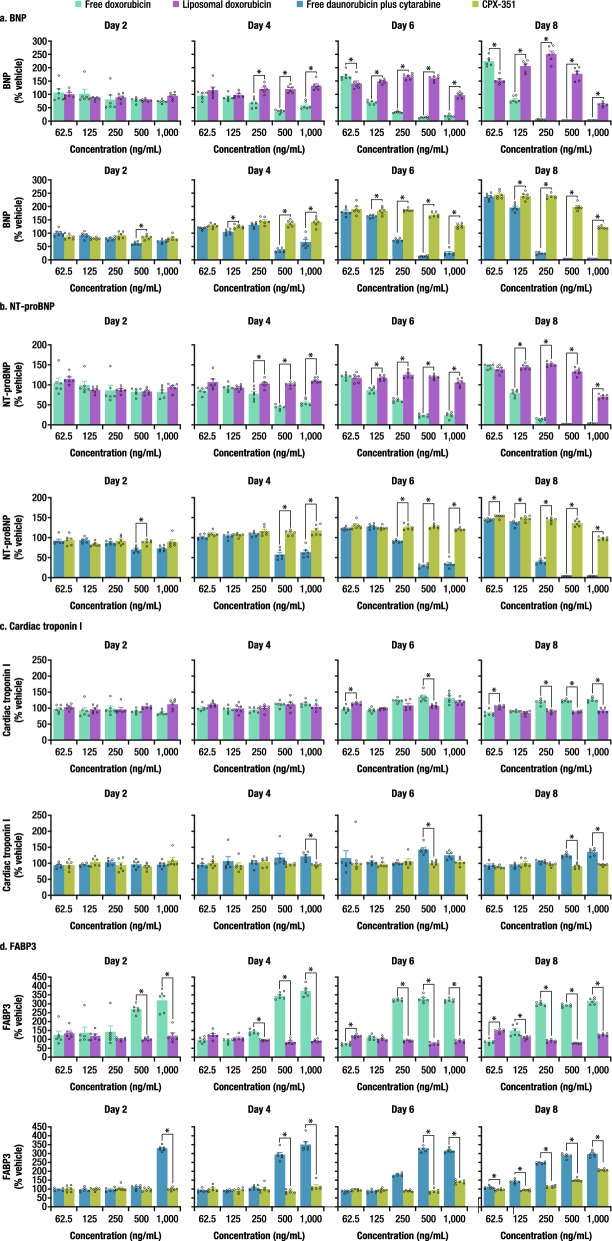
Cardiac biomarkers following treatment with free drugs versus liposomal formulations. (**a**) BNP. (**b**) NT-proBNP. (**c**) Cardiac troponin I. (**d**) FABP3. *BNP* brain natriuretic peptide, *FABP3* fatty acid–binding protein 3, *NT-proBNP* N-terminal pro–brain natriuretic peptide, *SEM* standard error of the mean. Data are mean ± SEM. The number of replicates was six for each assay. *Denotes q < 0.01, free versus liposomal.

Cardiac biomarkers were measured in the media on Days 2, 4, 6, and 8 using a multiplex assay. Intercellular adhesion molecule 1 (ICAM1) was not detected in the media (data not shown). Brain natriuretic peptide (BNP) and N-terminal proBNP (NT-proBNP), which are generally regarded as clinical biomarkers of heart failure^26^, followed similar patterns to each other (Fig. 4; Suppl. Table 2). For cells treated with free doxorubicin or daunorubicin (+ cytarabine), a concentration- and cumulative exposure-dependent decrease in the levels of BNP and NT-proBNP was observed; this decrease was likely due to poor cell health and/or viability at the evaluated concentrations. In contrast, for cells treated with the liposomal formulations, there was an increase (up to two times) in the levels of BNP and NT-proBNP by Day 8. Cardiac troponin I, a biomarker for the detection of cardiac injury^27^, was generally variable and did not display a clear response until Day 8, when a modest increase (~ 30%) was detected in cells treated with free doxorubicin at concentrations ≥ 250 ng/mL or daunorubicin (+ cytarabine) at concentrations ≥ 500 ng/mL but not with the liposomal formulations. Fatty acid-binding protein 3 (FABP3) release was consistently lower in cells treated with liposomal formulations versus free anthracyclines as early as Day 2 at the highest concentrations. Given the cell treatment cycles and biomarker sampling strategy, biomarker secretion peaks and troughs may have been missed due to media changes; therefore, these results should be interpreted with caution.

### Mitochondrial respiration is heavily compromised following treatment with free anthracyclines but not with liposomal anthracyclines

**Figure 5:**
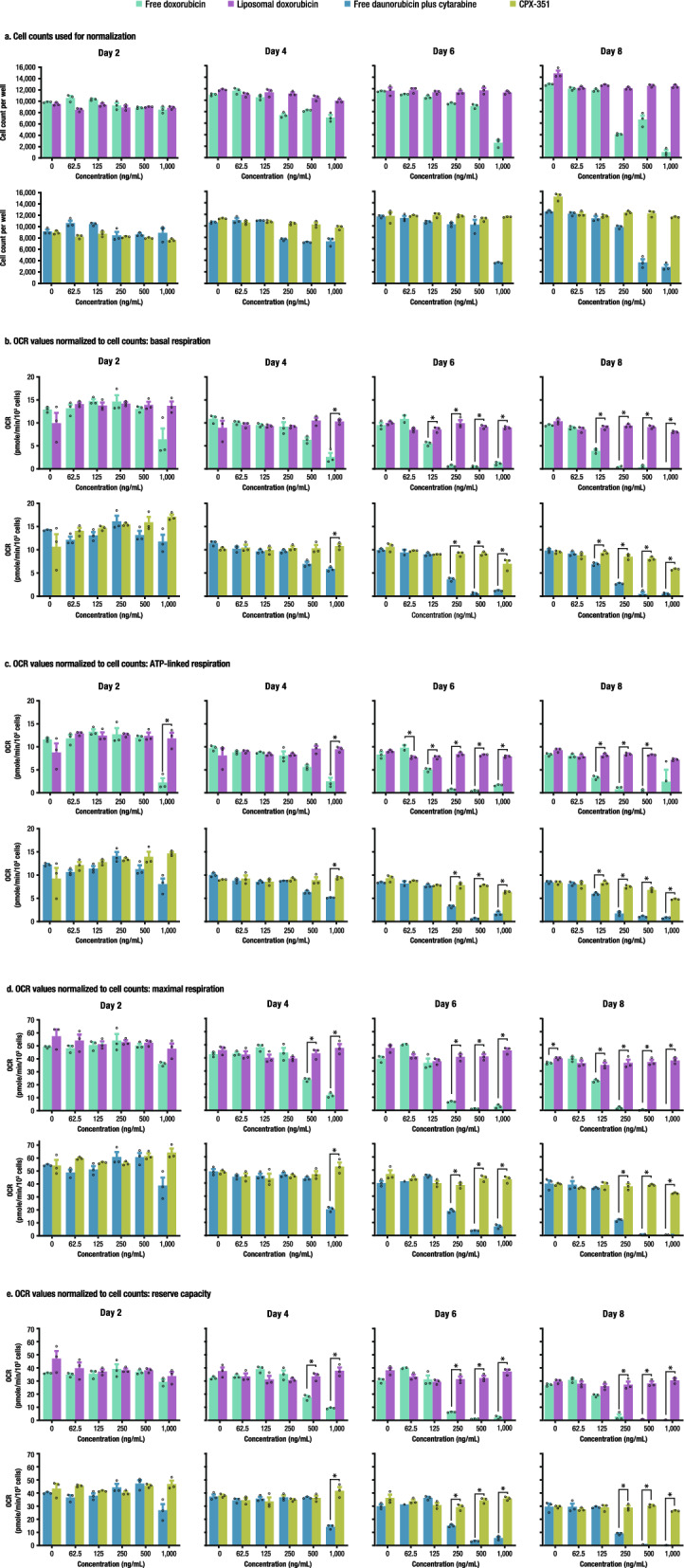
OCR values following treatment with free drugs versus liposomal formulations. (**a**) Cell counts used for normalization. (**b**) OCR values normalized to cell counts: basal respiration. (**c**) OCR values normalized to cell counts: ATP-linked respiration. (**d**) OCR values normalized to cell counts: maximal respiration. (**e**) OCR values normalized to cell counts: reserve capacity. *OCR* oxygen consumption rate; *ATP* adenosine triphosphate, *SEM* standard error of the mean. Cell counts were determined by Hoechst-positive nuclei. Data are mean ± SEM. The number of replicates was three for each assay. *Denotes q < 0.01, free versus liposomal.

The oxygen consumption rate (OCR) was assessed to evaluate the effect of anthracyclines on mitochondrial function. Parameters obtained from OCR measurements included basal respiration, ATP-linked respiration, maximal respiration, and reserve respiratory capacity. These parameters are all dependent on ATP-linked respiration to varying degrees, and our observations suggest impairment in ATP-linked respiration is the primary driver of the decline observed in mitochondrial function. However, a more generalized impairment of mitochondrial function could also be present. A decline in normalized OCR parameters was observed as early as Day 2 in cells treated with free doxorubicin or daunorubicin (+ cytarabine) at 1000 ng/mL (Fig. 5; Suppl. Table 2). On Day 4, the decline was more profound at 1000 ng/mL and was also noted at 500 ng/mL with both free doxorubicin and daunorubicin (+ cytarabine) for basal and ATP-linked respiration. By Day 6, the decline was observed at concentrations ≥ 250 ng/mL of free doxorubicin and daunorubicin (+ cytarabine). Finally, on Day 8, a decline in normalized OCR was observed at concentrations as low as 125 ng/mL in cells treated with doxorubicin or daunorubicin (+ cytarabine). There was some variability in the OCR of cells treated with liposomal doxorubicin or CPX-351, but no clear exposure-dependent inhibition of mitochondrial respiration was observed. Seahorse average time course traces are shown in Suppl. Fig. 1.

### hiPSC-derived cardiomyocyte uptake of daunorubicin

**Figure 6:**
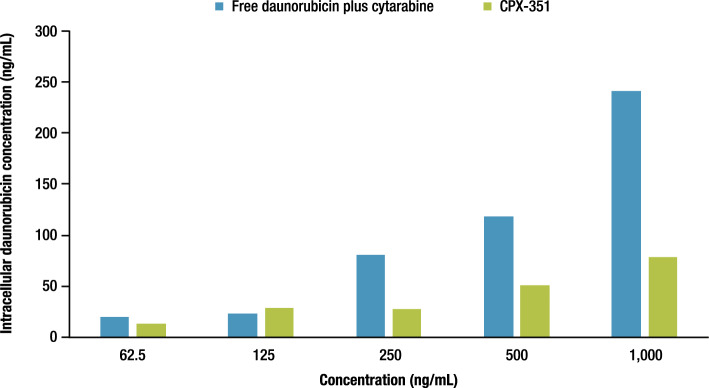
Intracellular daunorubicin concentration following treatment with free drugs versus CPX-351 liposomal formulation. Data available only as a single 24-h treatment.

Based on available data, a single 24-h treatment with 0 to 1000 ng/mL of daunorubicin (+ cytarabine) or CPX-351 was not expected to be cytotoxic. It was important for the cells to be alive to measure intracellular concentration; therefore, only a single 24-h treatment was performed to determine intracellular concentrations of daunorubicin. The intracellular concentrations of daunorubicin were comparable between treatments at lower concentrations; however, at concentrations ≥ 250 ng/mL, the total intracellular daunorubicin concentration was approximately two to three times lower in hiPSC-CM treated with CPX-351 than in those treated with the equivalent concentration of free drugs (Fig. 6). A caveat of the current method is that free intracellular concentrations of daunorubicin could not be determined and that free drug is likely the culprit for cardiotoxicity. It is noteworthy that different release profiles exist in different tissues for CPX-351^15^.

## Discussion

In this study, we used hiPSC-CM to investigate the relative cardiac safety profile of liposomal formulations of anthracyclines compared to their free-drug counterparts. Although it was not the intent of this work to directly compare doxorubicin and daunorubicin as cytarabine was included in both non-liposomal and liposomal conditions, overall, free doxorubicin appeared to be more cardiotoxic than daunorubicin by approximately a three-fold difference, which is consistent with clinical data^28^. Since clinical data demonstrating the cardioprotective effects of the liposomal encapsulation of doxorubicin already exist^12,29,30^, we used liposomal and free doxorubicin as prototypical compounds to establish proof of concept that the hiPSC-CM could distinguish between the two formulations and qualitatively recapitulate the clinical data in an in vitro context. When hiPSC-CM were treated with equivalent concentrations of doxorubicin, the model could discern the different cardiotoxicity potential of the liposomal formulation compared to the free drug. Since these preliminary results were promising, we next employed the model to evaluate whether CPX-351 (liposomal daunorubicin [+ cytarabine]) versus the free drugs would have the same benefits and included liposomal and free doxorubicin as positive controls. For all parameters assessed, the results for CPX-351 and free daunorubicin (+ cytarabine) were consistent with those observed with the prototypical compounds liposomal and free doxorubicin, respectively, indicating the liposomal formulation of CPX-351 is improving the cardiotoxicity profile of daunorubicin in this in vitro model. In this in vitro time-course study, comparison of measures of cellular health and function over the study duration suggested that the cardiotoxicity of the free anthracyclines increased upon repeated exposures (Suppl. Table 4).

Preliminary data from clinical studies also support a low risk of cardiotoxicity with CPX-351. In a pooled safety analysis of five clinical studies of CPX-351 in primarily older adults with newly diagnosed or relapsed/refractory AML, grade ≥ 3 cardiac adverse events were reported in 11% of patients treated with CPX-351 versus 14% of those treated with free chemotherapy combinations^31^. Although most of the pooled clinical studies did not evaluate cardiac events in detail, a phase 2 study of adults with acute leukemia found no clinically meaningful effects of CPX-351 treatment on heart rate or the QRS, PR, or QT intervals^32^. Additionally, in a separate phase 1/2 study in children with relapsed AML, only 1 in 38 patients developed a grade 3 reduction in ejection fraction, which subsequently resolved; a grade 2 reduction in ejection fraction occurred in six patients^33^.

Kopljar et al.^34^ also explored the potential of hiPSC-CM in detecting cardiac risk using doxorubicin as a prototypical compound. Consistent with their findings, our results suggest doxorubicin treatment resulted in an increase in cardiac troponin I and beat rate and a decrease in BNPs. Although Kopljar et al. used higher concentrations with a different treatment paradigm, the consistent results of these comparable studies clearly demonstrate the sensitivity of the model.

Unlike channel blockers, anthracyclines are not known to directly alter rhythmicity; however, sinus tachycardia and atrial and ventricular ectopic beats have been documented during anthracycline infusion^35^. Since hiPSC-CM have been shown to be useful for assessing this in vitro, and since proper rhythmicity is an important role of CM, this endpoint was included in our work^17,36^. During our initial assay development, other approaches, such as calcium flux and microelectrode array, were explored, but imaging data prove to be a reliable approach that could provide more information regarding CM function (data not shown). This approach has been recommended by the CiPA consortium^37^. Ultimately, we were able to show an increase in beat rate in vitro following anthracycline treatment when cells were relatively healthy and cell quiescence after multiple treatments, consistent with their compromised bioenergetics and viability. Both the cell quiescence and the reduced viability, as assessed by LDH release and ATP content, are consistent with progressive, cumulative toxicity. Importantly, metabolic impairment has been hypothesized as one of the underlying mechanisms in the failing heart^38^. In our work, we were able to show that free-anthracycline exposure rapidly and consistently resulted in mitochondrial dysfunction, which was consistent with the previous hypothesis that one of the mechanisms by which anthracyclines cause cumulative toxicity is via mitochondrial toxicity. Mitochondrial dysfunction would theoretically also result in reduced intracellular ATP, which is consistent with our observations.

Cardiac biomarker release in vitro is difficult to interpret due to the limitations associated with the sampling strategy with single collections on Days 2, 4, 6, and 8 and the fact that clinically, biomarkers of cardiac injury follow distinct patterns of release post injury and are loosely associated with different types of injury. Specifically, Troponin I is considered related to necrosis, FABP3 to ischemia, and NT-proBNP and BNP to hemodynamic changes. These later processes do not have a direct counterpart in a 2-dimensional in vitro model as used in this study. However, it is interesting to note that although the patterns of release did not necessarily mimic what would be expected clinically, the response was different between the liposomal and free-drug conditions, and the observations were qualitatively comparable between the doxorubicin-based formulas and the daunorubicin-based formulas, further indicating that CPX-351 has the potential to provide cardiac benefits to patients compared with the free-drug combo.

In the clinic, it has been hypothesized that cumulative exposure and C_max_ drive the cardiotoxicity of anthracyclines. Our results are consistent with these observations since higher toxicity was observed following exposure to higher concentrations and measured intracellular concentrations were also higher at these levels. In vivo, at the CM level, a higher C_max_ for free daunorubicin could result in higher intracellular concentrations of daunorubicin and likely more profound mitochondrial damage. Our previous in vivo PK data demonstrated a lower heart-to-plasma ratio in rats administered CPX-351 compared to the free-drug combination, which was also consistent with this hypothesis^15^.

A few limitations must be considered when evaluating the translational relevance of this study. First, while iCell hiPSC-CMs share many characteristics of normal human CMs, including metabolic profile, sarcomeric organization, cell–cell communication, and MLC2v expression, they also do beat spontaneously, which is more consistent with an immature, neonatal phenotype^39^. Another potential limitation of this study is that the drugs might have bound to the serum present in the media. For example, both doxorubicin and daunorubicin, as well as their main active metabolites (doxorubicinol and daunorubicinol), have been reported to bind to plasma proteins to levels of approximately 75%^3,40^. However, these limitations would not be expected to impact the outcomes of the study due to the strategic comparative study design, which used mirror conditions for each drug and formulation under study, and the fact that we were able to recapitulate the different cardiotoxicities that have been documented in the clinic with liposomal versus free doxorubicin, which provides further evidence of the relevance of the model.

Overall, we utilized an in vitro multiparametric model to assess the relative toxicity of anthracyclines across formulations using hiPSC-CM, which have been recognized in the field of cardiotoxicity safety assessment and have established proof of concept with compounds for which the clinical benefits have been demonstrated^12,29,30^. Then, we applied the same model to CPX-351 versus free daunorubicin (+ cytarabine). The results for CPX-351 versus free daunorubicin (+ cytarabine) were in accordance with those observed for liposomal versus free doxorubicin, suggesting comparable efficacy in preventing anthracycline-induced cardiotoxicity in hiPSC-CM. Liposomal formulations might provide important benefits, particularly to pediatric patients or older patients with previous anthracycline exposure or additional risk factors^28,41^. While the current study contributes to the overall weight of evidence, long-term clinical data are still needed to confirm the reduced cardiotoxicity observed with CPX-351 versus the free-drug combination in this in vitro model.

## Methods

### Cell culture

**Figure 7:**
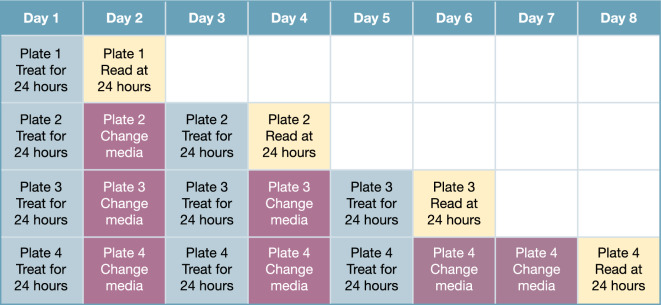
Dosing regimen of free drugs and liposomal formulations.

hiPSC-derived iCell-CM (Catalog # R1057, Cellular Dynamics, Madison, WI, USA; donor 01434: female, healthy, Caucasian, > 18 years old at sampling) were thawed and plated at 63,000 cells/cm^2^ into 384-well tissue culture-treated microplates (Corning, 3764, Corning, NY, USA) that had been pre-coated for 1 h with 0.1% gelatin (STEMCELL Technologies, 07903, Vancouver, Canada). Cultures were maintained for 7 days according to the manufacturer’s guidelines at 5% CO_2_ and 37 °C. After 7 days, doxorubicin (LC Laboratories, D-4000, Woburn, MA, USA), liposomal doxorubicin (FormuMax, F30204B-D, Sunnyvale, CA, USA), CPX-351 (Jazz Pharmaceuticals, Palo Alto, CA, USA), or a 1:5 molar ratio of free daunorubicin (SelleckChem, S3035, Houston, TX, USA) + cytarabine (SelleckChem, S1648) was applied to the CM for 24 h on Days 1, 3, and 5 at concentrations ranging from 0 to 1000 ng/mL (doxorubicin and daunorubicin) or 0 to 2273 ng/mL (cytarabine). Cells were maintained under 5% CO_2_ and 37 °C conditions and regular media changes with cardiac maintenance media were performed on non-dosing days (Days 2, 4, 6, and 7; Fig. 7). Plates were analyzed after one 24-h treatment period (Day 2), two 24-h treatment periods (Day 4), three 24-h treatment periods (Day 6), or three 24-h treatment periods plus a 48-h off-treatment period (Day 8) to assess recovery or delayed toxicities. Viability control compounds staurosporine (SelleckChem, S1421) and rotenone (Cayman Chemical, 13995, Ann Arbor, MI, USA) were prepared at six times their target concentrations (2 µM and 10 µM, respectively) and added (10 µL compound to 50 µL cells) to CM cultures 24 h prior to each assay time point. Vehicle control was 0.9% saline for all assays.

### Bright-field microscopy

Cell morphology and beating were assessed on Days 2, 4, 6, and 8 via bright-field microscopy using a Celldiscoverer 7 (Zeiss, Oberkochen, Germany) equipped with a 5 × 0.35 numerical aperture objective (1.822 µm/pixel). Imaging was performed under 5% CO_2_ and 37 °C conditions. Image acquisition was controlled by ZEN 2.6 software (Zeiss). Time-series images were acquired using camera streaming with a 30 ms exposure time at 3% transmitted light intensity over a 30-s window in a 512 × 512 pixel field of view at 28.3 frames per second (850 total images per series). All images were exported to .tif format for subsequent analysis. The first image in each series was used to qualitatively assess culture morphology. The full-time series images were used for quantitative analysis of CM beating characteristics.

### Intracellular ATP

After media collection and functional assessment, hiPSC-CM plates were rinsed 6six times with phosphate-buffered saline (PBS)+/+ (Corning, 21–030-CV), leaving 25 µL/well PBS+/+. The CellTiter-Glo 3D Cell Viability Assay (Promega, G9682, Madison, WI, USA) was used to assess changes in ATP content in CM cultures according to the manufacturer’s guidelines. Briefly, after thawing and equilibrating the CellTiter-Glo reagent to room temperature, 25 µL/well of the CellTiter-Glo 3D reagent was added to the plate, followed by a 5-min shake step and a 20-min room-temperature incubation. Shake steps and luminescence recordings were accomplished using an EnVision plate reader (PerkinElmer, Waltham, MA, USA). Data were analyzed and plotted using GraphPad Prism as relative luminescence units.

### LDH release

The LDH-Glo Cytotoxicity Assay (Promega, J2380) was used to assess LDH release from hiPSC-CM cultures according to the manufacturer’s guidelines. Briefly, an LDH storage buffer (200 mM Tris–HCl, 10% glycerol, 1% bovine serum albumin) was prepared in advance of all assays and stored at 4 °C for up to 1 month. Prior to functional assessment, 10 µL/well of media were collected and diluted 1:30 in an LDH storage buffer. This diluted sample was then combined with a freshly prepared detection mix (12 µL reductase substrate + 2.5 mL enzyme mix) in a 1:1 ratio (10 µL + 10 µL). This detection mix was also added to a freshly prepared LDH standard curve at LDH concentrations ranging from 0 to 32 mU/mL (provided with the kit). After a 30-min room-temperature incubation, luminescence values were measured using an EnVision plate reader. Data were converted to absolute LDH concentration in Excel and plotted using GraphPad Prism as mU/mL.

### Image analysis

Time-series images were analyzed using ImageJ (National Institutes of Health, Bethesda, MD, USA). Image series were first filtered by subtraction of a Gaussian-blurred (σ = 10 pixels) copy of the original stack, then stabilized using the Image Stabilizer plugin^42^. Fifty frames of the median image of each time series were then appended to the beginning of the image stack, creating a reference frame for subsequent beating analysis using the MUSCLEMOTION plugin in batch-processing mode^43^. Based on analysis of positive control (vehicle-treated) and negative control (staurosporine-treated) wells, the following options were selected for MUSCLEMOTION analysis: Gaussian blur—no; frame rate—28.3; speedWindow—2; decrease noise—yes, simple; detect reference frame—yes, simple; and analyze transients—yes, simple. Time-series analysis outputs (overview-results.txt files) were compiled and filtered to true contractile events (peak amplitude > 90, contractile amplitude > 1/3 vehicle control–treated wells) using JMP 14.0.0 (SAS, Cary, NC, USA), then exported and plotted as beat rate using GraphPad Prism.

### Cardiotoxicity biomarker assessment

On Days 2, 4, 6, and 8, 30 µL/well of media were collected in 384-well v-bottom plates (Greiner Bio-One, 781281, Monroe, NC, USA), split into two replicates (one kept as a backup), and frozen at –80 °C. At the completion of the study, media samples were shipped to Abcam for FirePlex cardiotoxicity immunoprofiling (Abcam, ab252375, Waltham, MA, USA) to quantify cardiac biomarkers (ICAM1, cardiac troponin I, FABP3, BNP, and NT-proBNP). Data were analyzed in Excel and plotted using GraphPad Prism as pg/mL. Values less than the minimal detectable dose (MDD) were set to the MDD (8.8, 102.6, 1.9, 10.4, and 10.3 pg/mL for ICAM1, cardiac troponin I, FABP3, BNP, and NT-proBNP, respectively). Values greater than the upper limit of quantitation (uLOQ) were set to the uLOQ (30,000, 30,000, 1111, 3333, and 3333 pg/mL for ICAM1, cardiac troponin I, FABP3, BNP, and NT-proBNP, respectively). Raw fluorescence data were normalized to vehicle control in Excel and plotted using GraphPad Prism as percent change. Each biomarker had a unique time course following myocardial injury.

### Mitochondrial respiration

The metabolic profiles were generated using the Seahorse XF96 platform (Agilent, Santa Clara, CA, USA) by measuring OCRs. Briefly, 15,000 hiPSC-CM were seeded in each well of a XF96-well cell culture microplate, allowed to recover, and treated, as described above. The assay medium (Seahorse DMEM supplemented with 4 mM of l-glutamine, 10 mM of galactose, and 1 mM of sodium pyruvate) was pre-warmed and adjusted to pH 7.4. Plates were washed with the assay medium three times, a final volume of 175 μL of the assay medium was added, and plates were incubated at 37 °C without CO_2_ for 60 min prior to loading into the XF96 Extracellular Flux Analyzer. The mitochondrial stress test conditions included the following sequential injections: oligomycin (25 μL at 20 μM to a final concentration of 2.5 μM), carbonyl cyanide-4-(trifluoromethoxy)phenylhydrazone (FCCP; 25 μL at 18 μM to a final concentration of 2 μM), FCCP (25 μL at 18 μM to a final concentration of 3.6 μM), and rotenone/antimycin A (25 μL at 22 μM to a final concentration of 2 μM). Upon completion of each respirometry assay, the XF96 microplate was removed and fixed with 4% paraformaldehyde. After fixation, the cells were stained with Hoechst, and the cell number per well was assessed via high-content imaging. The respirometry well-level data (pmoles O_2_/min) were normalized per cell number (pmoles O_2_/min/103 cells) in each independent experiment. Following normalization of well data based on well cell counts, the non-mitochondrial respiration was subtracted from the other parameters to assess the following aspects of mitochondrial respiration: basal respiration, ATP-linked respiration, maximal respiration, and reserve respiratory capacity. Details regarding the different aspects of mitochondrial respiration are provided in Suppl. Table 1.

### Intracellular daunorubicin concentrations

To evaluate if the differences observed between daunorubicin (+ cytarabine) and CPX-351 were due to differential uptake of liposomal versus free anthracyclines, hiPSC-CM were treated with 0 to 1000 ng/mL of daunorubicin (+ cytarabine) and CPX-351 for 24 h. Cells were then rinsed twice with PBS, dissociated, pooled at 6 wells per sample using pre-warmed TrypLE (Gibco, 12605010, Thermo Fisher Scientific, Waltham, MA, USA), and lysed using 100 μL per sample CelLytic MT Buffer (Sigma-Aldrich, C3228, St. Louis, MO, USA). The total protein concentration was assessed via Pierce BCA assay (Thermo Fisher Scientific, 23227) with absorbance measurements at 562 nm acquired on an EnVision (PerkinElmer) plate reader. Lysate supernatant was snap frozen and analyzed for total daunorubicin content.

A high-performance liquid chromatography system (Shimadzu, LC-20, Kyoto, Japan) connected with triple quadrupole mass spectrometer (Sciex, API 4500, Framingham, MA, USA) was used to measure the daunorubicin in CM cell lysate. A standard curve with nine points at 1, 2, 5, 10, 50, 100, 500, 800, and 1000 ng/mL and four quality control levels at 3, 30, 300, and 750 ng/mL were used in the assay. 25 μL of each standard, quality control, sample, and blank were added into the plate according to the analytical run sheet. Daunorubicin-^13^C,d_3_ was used as the internal standard and prepared at 25 ng/mL in methanol with 0.5% trifluoroacetic acid as the internal standard solution. 200 μL of the internal standard solution was added to each sample and vortexed at 1500 rpm for 5 min. The sample plate was then centrifuged at 3000×*g* for 10 min under refrigerated conditions. The supernatant (150 μL) was transferred into a new plate. Fifty (50) μL of methanol with 1% NH_4_OH was added to each well. The plate was vortexed and centrifuged and ready for injection.

Chromatographic separation was carried out with a Waters AMIDE column (2.5 μM, 2.1 × 100 mm). Mobile phase A was 10 mM ammonium formate with 0.1% NH_4_OH in water, and mobile phase B was 10 mM ammonium formate with 0.1% NH_4_OH in acetonitrile/water (90/10). The gradient for mobile phase B was 100% from 0 to 0.5 min, decreased to 60% at 2 min, kept at 60% until 3 min, quickly increased to 100% at 3.1 min, and stopped at 6 min. The follow rate was kept at 0.5 mL/min, and column temperature was set at 60 °C. A multiple reaction monitoring transition of 528.2 to 321.1 was chosen for daunorubicin and 532.2 to 325.1 was chosen for Daunorubicin-^13^C,d_3_.

### Statistical analyses

Data were compiled, analyzed, and graphed using Microsoft Excel and GraphPad Prism 9. For the biological endpoints of intracellular ATP, LDH release, beat rate, contractile amplitude, mechanical output, and cardiac biomarkers, the number of replicates was six. All values, except LDH concentration, were expressed as a percent of vehicle control cells. For the oxygen consumption rate, the number of replicates was three and the data from each well were individually normalized based on the number of cells present within that well (based on Hoechst-stained nuclei counts). To assess the effect of repeated exposure/time and concentration on a given parameter within a specific treatment (e.g., the effect of the analysis Day [2, 4, 6, or 8] and concentration [62.5–1000 ug/mL] on basal respiration of cells treated with free doxorubicin), two-way analysis of variance (ANOVA) was used with Tukey’s multiple comparison test to assess the effect of concentration and the effect of the Day of assessment, with q-values less than 0.01 considered statistically significant. The repeated-measures ANOVA was not deemed appropriate since they were not consecutive samples from the same biological entity. To further evaluate whether liposomal encapsulation of the drugs had an impact at any given concentration and on any given day, multiple unpaired t-tests using two-stage step-up (Benjamini, Krieger, and Yekutieli) with a false discovery rate of 1% to account for the multiple comparisons were conducted between the two treatment groups for each endpoint (e.g., the effect on LDH release with treatment of 500 ug/mL free versus liposomal doxorubicin on Day 4). Only values considered to be discoveries are reported as significant (i.e., q-values < 0.01).

### Supplementary Information

Supplementary Information.

## Data Availability

All relevant data are provided with the manuscript and supporting files.
