# Supplementary material for: Lower cardiotoxicity of CPX-351 relative to daunorubicin plus cytarabine free-drug combination in hiPSC-derived cardiomyocytes in vitro
Source: Sci Rep. 2023 Nov 29;13:21054. doi: 10.1038/s41598-023-47293-4 (PMC10686991; doi:10.1038/s41598-023-47293-4)
Supplement: Supplementary file 1 — Supplementary Information. [file 41598_2023_47293_MOESM1_ESM.docx]

**Supplementary Information

Lower cardiotoxicity of CPX-351 relative to daunorubicin plus cytarabine free-drug combination in hiPSC-derived cardiomyocytes *in vitro***

Marie C. Fortin,^1,^* Andrew S. LaCroix,^2^ Tom N. Grammatopoulos,^3^ Lei Tan,^1^ Qi Wang,^1^ Dino Manca^1^

**Supplementary Table 1.** Mitochondrial respiration.

| **Basal respiration** | The oxygen consumption used to meet cellular ATP demand and resulting from mitochondrial proton leak. Shows energetic demand of the cell under baseline conditions |
| --- | --- |
| **ATP-linked respiration** | The decrease in OCR upon injection of the ATP synthase inhibitor oligomycin represents the portion of basal respiration that was being used to drive ATP production. Shows ATP produced by the mitochondria that contribute to meeting the energetic needs of the cell |
| **Maximal respiration** | The maximal OCR attained by adding the uncoupler FCCP. FCCP mimics a physiological “energy demand” by stimulating the respiratory chain to operate at maximum capacity, which causes rapid oxidation of substrates (sugars, fats, and amino acids) to meet this metabolic challenge. Shows the maximum rate of respiration that the cell can achieve |
| **Reserve respiratory capacity** | This measurement indicates the capability of the cell to respond to an energetic demand as well as how close the cell is to respiring to its theoretical maximum. The cell’s ability to respond to demand can be an indicator of cell fitness or flexibility |

ATP, adenosine triphosphate; OCR, oxygen consumption rate; FCCP, carbonyl cyanide-4-(trifluoromethoxy)phenylhydrazone.

**Supplementary Table 2.** Summary of the significant data points (q <0.01) from a) Figure 2, b) Figure 4, and c) Figure 5, including the means and q-values.

a)

|  | **Concentration (ng/mL)** | **Free versus liposomal doxorubicin** | | **Free daunorubicin plus cytarabine versus CPX-351** | |
| --- | --- | --- | --- | --- | --- |
|  |  | **Mean vs Mean** | **q-value** | **Mean vs Mean** | **q-value** |
| **Figure 2a. ATP content – CTG signal (% vehicle)** | | | | | |
| Day 2 | 250 | 72.22 vs 96.67 | <0.000001 | - | - |
| Day 2 | 500 | 80.93 vs 93.65 | 0.001017 | 55.27 vs 95.25 | <0.000001 |
| Day 4 | 62.5 | 83.22 vs 91.08 | 0.000768 | - | - |
| Day 4 | 125 | 67.93 vs 88.92 | <0.000001 | 77.88 vs 87.82 | 0.000014 |
| Day 4 | 250 | 80.90 vs 87.88 | 0.000768 | 70.40 vs 86.58 | <0.000001 |
| Day 4 | 500 | 56.72 vs 86.12 | <0.000001 | 71.45 vs 84.95 | <0.000001 |
| Day 4 | 1,000 | - | - | 70.87 vs 86.18 | 0.000008 |
| Day 6 | 125 | 65.70 vs 82.27 | 0.000002 | - | - |
| Day 6 | 250 | 32.93 vs 81.03 | <0.000001 | 59.23 vs 82.92 | <0.000001 |
| Day 6 | 500 | 14.68 vs 81.77 | <0.000001 | 16.07 vs 81.47 | <0.000001 |
| Day 6 | 1,000 | 26.53 vs 71.83 | <0.000001 | 22.83 vs 76.28 | <0.000001 |
| Day 8 | 125 | 68.15 vs 80.38 | 0.000006 | - | - |
| Day 8 | 250 | 7.133 vs 82.65 | <0.000001 | 48.35 vs 76.75 | <0.000001 |
| Day 8 | 500 | 0.6000 vs 77.05 | <0.000001 | 0.5000 vs 71.85 | <0.000001 |
| Day 8 | 1,000 | 1.933 vs 63.28 | <0.000001 | 1.883 vs 61.50 | <0.000001 |
| **Figure 2b. LDH release (mU/mL)** | | | | | |
| Day 2 | 500 | 0.3009 vs 0.1960 | 0.000024 | - | - |
| Day 2 | 1,000 | 0.3316 vs 0.2181 | 0.000328 | 0.3491 vs 0.1957 | 0.000006 |
| Day 4 | 250 | 0.4007 vs 0.2583 | 0.000134 | - | - |
| Day 4 | 500 | 2.444 vs 0.2592 | <0.000001 | 1.287 vs 0.2677 | <0.000001 |
| Day 4 | 1,000 | 2.222 vs 0.2495 | <0.000001 | 2.148 vs 0.3207 | <0.000001 |
| Day 6 | 62.5 | 0.1285 vs 0.1641 | 0.000391 | - | - |
| Day 6 | 125 | 0.1786 vs 0.1604 | 0.009039 | - | - |
| Day 6 | 250 | 1.750 vs 0.1436 | <0.000001 | 0.3127 vs 0.1479 | 0.000002 |
| Day 6 | 500 | 2.140 vs 0.1373 | <0.000001 | 2.639 vs 0.1450 | <0.000001 |
| Day 6 | 1,000 | 2.175 vs 0.1478 | <0.000001 | 2.660 vs 0.2341 | <0.000001 |
| Day 8 | 62.5 | 0.3050 vs 0.4280 | 0.000002 | 0.3874 vs 0.3369 | 0.000559 |
| Day 8 | 125 | - | - | 0.5010 vs 0.3258 | 0.000016 |
| Day 8 | 250 | 3.127 vs 0.3281 | 0.000001 | 1.432 vs 0.3993 | <0.000001 |
| Day 8 | 500 | 2.120 vs 0.3030 | <0.000001 | 3.048 vs 0.5330 | <0.000001 |
| Day 8 | 1,000 | 3.400 vs 0.4154 | <0.000001 | 4.136 vs 0.9056 | <0.000001 |

b)

|  | **Concentration (ng/mL)** | **Free versus liposomal doxorubicin** | | **Free daunorubicin plus cytarabine versus CPX-351** | |
| --- | --- | --- | --- | --- | --- |
|  |  | **Mean vs Mean** | **q-value** | **Mean vs Mean** | **q-value** |
| **Figure 4a. BNP (% vehicle)** | | | | | |
| Day 2 | 500 | - | - | 60.23 vs 84.06 | 0.001044 |
| Day 4 | 125 | - | - | 103.5 vs 124.1 | 0.001263 |
| Day 4 | 250 | 67.16 vs 120.5 | 0.000246 | - | - |
| Day 4 | 500 | 35.66 vs 119.7 | <0.000001 | 34.51 vs 136.2 | <0.000001 |
| Day 4 | 1,000 | 58.12 vs 132.1 | 0.000016 | 65.84 vs 140.8 | 0.000080 |
| Day 6 | 62.5 | 169.4 vs 141.2 | 0.006955 | - | - |
| Day 6 | 125 | 69.56 vs 148.6 | <0.000001 | 162.9 vs 183.5 | 0.001643 |
| Day 6 | 250 | 32.65 vs 167.4 | <0.000001 | 72.95 vs 186.2 | <0.000001 |
| Day 6 | 500 | 12.47 vs 160.4 | <0.000001 | 11.69 vs 168.5 | <0.000001 |
| Day 6 | 1,000 | 17.13 vs 96.81 | <0.000001 | 25.45 vs 127.2 | <0.000001 |
| Day 8 | 62.5 | 225.0 vs 152.5 | 0.000022 | - | - |
| Day 8 | 125 | 80.14 vs 206.3 | <0.000001 | 195.6 vs 239.5 | 0.000081 |
| Day 8 | 250 | 5.184 vs 251.1 | <0.000001 | 23.20 vs 238.4 | <0.000001 |
| Day 8 | 500 | 1.606 vs 177.4 | <0.000001 | 2.007 vs 198.2 | <0.000001 |
| Day 8 | 1,000 | 2.297 vs 62.40 | <0.000001 | 2.527 vs 120.6 | <0.000001 |
| **Figure 4b. NT-proBNP (% vehicle)** | | | | | |
| Day 2 | 500 | - | - | 70.34 vs 89.01 | 0.002475 |
| Day 4 | 250 | 77.91 vs 102.9 | 0.006949 | - | - |
| Day 4 | 500 | 44.01 vs 101.0 | <0.000001 | 57.97 vs 112.5 | 0.000007 |
| Day 4 | 1,000 | 56.22 vs 110.9 | <0.000001 | 63.67 vs 118.2 | 0.000047 |
| Day 6 | 125 | 87.21 vs 117.1 | 0.000004 | - | - |
| Day 6 | 250 | 60.83 vs 125.3 | <0.000001 | 90.79 vs 126.1 | <0.000001 |
| Day 6 | 500 | 22.71 vs 119.7 | <0.000001 | 29.64 vs 127.4 | <0.000001 |
| Day 6 | 1,000 | 23.83 vs 106.0 | <0.000001 | 34.45 vs 120.4 | <0.000001 |
| Day 8 | 62.5 | - | - | 144.6 vs 152.9 | 0.002698 |
| Day 8 | 125 | 79.36 vs 144.6 | <0.000001 | 136.0 vs 147.9 | 0.008152 |
| Day 8 | 250 | 13.67 vs 151.3 | <0.000001 | 40.40 vs 144.2 | <0.000001 |
| Day 8 | 500 | 1.872 vs 133.1 | <0.000001 | 3.843 vs 136.6 | <0.000001 |
| Day 8 | 1,000 | 3.281 vs 70.29 | <0.000001 | 3.901 vs 97.65 | <0.000001 |
| **Figure 4c. Cardiac troponin I (% vehicle)** | | | | | |
| Day 4 | 1,000 | - | - | 121.3 vs 94.46 | 0.003276 |
| Day 6 | 62.5 | 98.32 vs 115.0 | 0.003909 | - | - |
| Day 6 | 500 | 135.5 vs 107.7 | 0.003909 | 142.6 vs 100.4 | 0.000676 |
| Day 8 | 62.5 | 84.80 vs 105.8 | 0.000142 | - | - |
| Day 8 | 250 | 120.7 vs 90.92 | 0.000009 | - | - |
| Day 8 | 500 | 123.5 vs 88.93 | <0.000001 | 124.5 vs 91.02 | 0.000026 |
| Day 8 | 1,000 | 126.7 vs 92.85 | 0.000004 | 135.4 vs 94.97 | 0.000020 |
| **Figure 4d. FABP3 (% vehicle)** | | | | | |
| Day 2 | 500 | 269.3 vs 101.2 | <0.000001 | - | - |
| Day 2 | 1,000 | 319.3 vs 120.4 | 0.000076 | 329.3 vs 99.79 | <0.000001 |
| Day 4 | 250 | 140.6 vs 94.96 | 0.000006 | - | - |
| Day 4 | 500 | 345.4 vs 85.63 | <0.000001 | 293.6 vs 85.27 | <0.000001 |
| Day 4 | 1,000 | 372.4 vs 91.39 | <0.000001 | 351.1 vs 114.3 | <0.000001 |
| Day 6 | 62.5 | 73.87 vs 118.6 | <0.000001 | - | - |
| Day 6 | 250 | 322.2 vs 91.68 | <0.000001 | 180.7 vs 89.45 | <0.000001 |
| Day 6 | 500 | 327.1 vs 76.92 | <0.000001 | 324.0 vs 87.27 | <0.000001 |
| Day 6 | 1,000 | 319.5 vs 89.69 | <0.000001 | 316.5 vs 140.3 | <0.000001 |
| Day 8 | 62.5 | 85.71 vs 146.7 | 0.000001 | 109.2 vs 96.03 | 0.007902 |
| Day 8 | 125 | 148.5 vs 110.0 | 0.003132 | 141.9 vs 93.22 | 0.000002 |
| Day 8 | 250 | 300.1 vs 90.22 | <0.000001 | 246.8 vs 113.8 | <0.000001 |
| Day 8 | 500 | 290.1 vs 77.85 | <0.000001 | 287.7 vs 149.7 | <0.000001 |
| Day 8 | 1,000 | 314.9 vs 124.4 | <0.000001 | 297.1 vs 207.2 | <0.000001 |

c)

|  | **Concentration (ng/mL)** | **Free versus liposomal doxorubicin** | | **Free daunorubicin plus cytarabine versus CPX-351** | |
| --- | --- | --- | --- | --- | --- |
|  |  | **Mean vs Mean** | **q-value** | **Mean vs Mean** | **q-value** |
| **Figure 5b. OCR values normalized to cell counts: basal respiration (pmol/min/10^3^ cells)** | | | | | |
| Day 4 | 1,000 | 2.548 vs 10.24 | 0.005592 | 5.800 vs 10.76 | 0.003993 |
| Day 6 | 125 | 5.430 vs 8.495 | 0.001101 | - | - |
| Day 6 | 250 | 0.5654 vs 9.875 | 0.000105 | 3.588 vs 9.090 | 0.000173 |
| Day 6 | 500 | 0.3429 vs 9.093 | 0.000008 | 0.4824 vs 9.113 | 0.000025 |
| Day 6 | 1,000 | 1.081 vs 8.883 | 0.000008 | 1.127 vs 6.956 | 0.001549 |
| Day 8 | 125 | 3.862 vs 8.930 | 0.000164 | 6.876 vs 9.323 | 0.000963 |
| Day 8 | 250 | -0.8097 vs 9.318 | 0.000554 | 2.652 vs 8.496 | 0.000070 |
| Day 8 | 500 | 0.2909 vs 9.020 | 0.000024 | 0.4297 vs 8.035 | 0.000070 |
| Day 8 | 1,000 | -0.7483 vs 7.966 | 0.000024 | 0.4398 vs 5.758 | 0.000014 |
| **Figure 5c. OCR** **values normalized to cell counts: ATP-linked respiration (pmol/min/10^3^ cells)** | | | | | |
| Day 4 | 1,000 | 2.384 vs 9.424 | 0.007037 | 5.100 vs 9.386 | 0.000092 |
| Day 6 | 62.5 | 9.774 vs 7.663 | 0.008194 | - | - |
| Day 6 | 125 | 4.944 vs 7.693 | 0.000474 | - | - |
| Day 6 | 250 | 0.6087 vs 8.368 | 0.000008 | 3.205 vs 7.824 | 0.000368 |
| Day 6 | 500 | 0.3751 vs 8.172 | 0.000001 | 0.5689 vs 7.717 | 0.000003 |
| Day 6 | 1,000 | 1.588 vs 7.839 | 0.000003 | 1.695 vs 6.328 | 0.000211 |
| Day 8 | 125 | 3.233 vs 8.119 | 0.000181 | 5.936 vs 8.309 | 0.000879 |
| Day 8 | 250 | 0.6992 vs 8.366 | 0.000175 | 1.716 vs 7.470 | 0.000055 |
| Day 8 | 500 | -0.07900 vs 8.177 | 0.000175 | 1.026 vs 6.769 | 0.000037 |
| Day 8 | 1,000 | - | - | 0.7649 vs 4.753 | 0.000007 |
| **Figure 5d. OCR values normalized to cell counts: maximal respiration (pmol/min/10^3^ cells)** | | | | | |
| Day 4 | 500 | 23.84 vs 44.10 | 0.002443 | - | - |
| Day 4 | 1,000 | 11.85 vs 48.21 | 0.001260 | 19.97 vs 52.48 | 0.002936 |
| Day 6 | 250 | 6.845 vs 41.40 | 0.000049 | 18.64 vs 38.33 | 0.000257 |
| Day 6 | 500 | 1.429 vs 41.56 | 0.000032 | 3.703 vs 43.57 | 0.000028 |
| Day 6 | 1,000 | 3.175 vs 46.30 | 0.000032 | 6.791 vs 42.74 | 0.000058 |
| Day 8 | 0 | 36.53 vs 40.19 | 0.003612 | - | - |
| Day 8 | 125 | 22.92 vs 35.11 | 0.001558 | - | - |
| Day 8 | 250 | 2.056 vs 36.73 | 0.000118 | 11.60 vs 37.43 | 0.000119 |
| Day 8 | 500 | 0.4080 vs 37.34 | 0.000030 | 0.4237 vs 38.38 | <0.000001 |
| Day 8 | 1,000 | -1.611 vs 38.61 | 0.000030 | 0.4268 vs 32.10 | <0.000001 |
| **Figure 5e. OCR values normalized to cell counts: reserve capacity (pmol/min/10^3^ cells)** | | | | | |
| Day 4 | 500 | 17.56 vs 33.66 | 0.003694 | - | - |
| Day 4 | 1,000 | 9.298 vs 37.97 | 0.001526 | 14.17 vs 41.72 | 0.004071 |
| Day 6 | 250 | 6.279 vs 31.52 | 0.000224 | 15.05 vs 29.24 | 0.000792 |
| Day 6 | 500 | 1.086 vs 32.47 | 0.000063 | 3.221 vs 34.46 | 0.000028 |
| Day 6 | 1,000 | 2.095 vs 37.42 | 0.000063 | 5.664 vs 35.78 | 0.000028 |
| Day 8 | 250 | 2.865 vs 27.41 | 0.001016 | 8.949 vs 28.94 | 0.000185 |
| Day 8 | 500 | 0.1170 vs 28.32 | 0.000089 | -0.006006 vs 30.34 | 0.000003 |
| Day 8 | 1,000 | -0.8623 vs 30.64 | 0.000089 | -0.01293 vs 26.34 | <0.000001 |
| ATP, adenosine triphosphate; BNP, brain natriuretic peptide; CTG, CellTiter-Glo^®^; FABP3, fatty acid–binding protein 3; LDH, lactate dehydrogenase; NT-proBNP, N-terminal pro–brain natriuretic peptide; OCR, oxygen consumption rate. | | | | | |

**Supplementary Table 3.** Mean absolute beat rate (beats per minute) following treatment with free drugs versus liposomal formulations.

| **Mean beat rate (beats per minute)** | **CPX-351** | **Free daunorubicin (+ cytarabine)** | **Liposomal doxorubicin** | **Free doxorubicin** |
| --- | --- | --- | --- | --- |
|  |  |  |  |  |
| Day 2 |  |  |  |  |
| Control | 27.00 | | | |
| 62.5 | 31.00 | 35.33 | 28.67 | 38.33 |
| 125 | 31.33 | 41.33 | 27.67 | 49.00 |
| 250 | 32.33 | 51.00 | 29.00 | 43.00 |
| 500 | 34.33 | 41.00 | 29.33 | 40.00 |
| 1,000 | 38.67 | 41.00 | 32.33 | 37.67 |
| Day 4 |  |  |  |  |
| Control | 29.83 | | | |
| 62.5 | 31.33 | 32.00 | 27.33 | 32.33 |
| 125 | 33.33 | 36.33 | 28.33 | 42.67 |
| 250 | 34.33 | 50.33 | 30.00 | 9.667 |
| 500 | 38.00 | 30.67 | 32.33 | 64.33 |
| 1,000 | 41.67 | 66.67 | 37.00 | 68.00 |
| Day 6 |  |  |  |  |
| Control | 28.83 | | | |
| 62.5 | 31.00 | 34.00 | 27.00 | 33.67 |
| 125 | 32.67 | 43.67 | 28.33 | 33.33 |
| 250 | 36.67 | 39.67 | 30.00 | 32.00 |
| 500 | 40.33 | 38.00 | 33.33 | 34.00 |
| 1,000 | 37.00 | 38.33 | 33.00 | 13.00 |
| Day 8 |  |  |  |  |
| Control | 24.67 | | | |
| 62.5 | 31.67 | 35.33 | 27.67 | 35.33 |
| 125 | 34.00 | 46.00 | 28.67 | 13.67 |
| 250 | 38.33 | 7.333 | 30.67 | 10.33 |
| 500 | 40.67 | 0.000 | 32.33 | 15.33 |
| 1,000 | 35.67 | 6.000 | 30.67 | 11.33 |

**Supplementary Table 4.** Results of a two-way ANOVA using Tukey’s multiple comparisons test for all endpoints.

|  | **CPX351** | | **Free daunorubicin + cytarabine** | | **Liposomal doxorubicin** | | **Free doxorubicin** | |
| --- | --- | --- | --- | --- | --- | --- | --- | --- |
|  | % of total variation | P value | % of total variation | P value | % of total variation | P value | % of total variation | P value |
| **CTG signal** |  |  |  |  |  |  |  |  |
| Interaction | 7.292 | <0.0001 | 22.37 | <0.0001 | 7.386 | <0.0001 | 22.57 | <0.0001 |
| Concentration | 10.88 | <0.0001 | 45.05 | <0.0001 | 16.43 | <0.0001 | 27.59 | <0.0001 |
| Day | 66.11 | <0.0001 | 31.62 | <0.0001 | 65.52 | <0.0001 | 48.22 | <0.0001 |
| **LDH release** |  |  |  |  |  |  |  |  |
| Interaction | 26.96 | <0.0001 | 21.94 | <0.0001 | 5.426 | <0.0001 | 24.10 | <0.0001 |
| Concentration | 13.73 | <0.0001 | 51.09 | <0.0001 | 4.285 | <0.0001 | 42.45 | <0.0001 |
| Day | 55.95 | <0.0001 | 25.32 | <0.0001 | 84.19 | <0.0001 | 26.58 | <0.0001 |
| **Beat rate** |  |  |  |  |  |  |  |  |
| Interaction | 9.001 | 0.0163 | 39.25 | <0.0001 | 10.50 | <0.0001 | 32.23 | <0.0001 |
| Concentration | 27.98 | <0.0001 | 7.857 | 0.0001 | 38.12 | <0.0001 | 5.920 | 0.0060 |
| Day | 29.14 | <0.0001 | 21.74 | <0.0001 | 34.10 | <0.0001 | 23.38 | <0.0001 |
| **Contractile amplitude** |  |  |  |  |  |  |  |  |
| Interaction | 5.214 | 0.7692 | 24.29 | <0.0001 | 28.38 | <0.0001 | 47.29 | <0.0001 |
| Concentration | 17.88 | <0.0001 | 28.18 | <0.0001 | 9.801 | 0.0043 | 10.76 | <0.0001 |
| Day | 12.79 | 0.0004 | 26.88 | <0.0001 | 1.459 | 0.4935 | 15.08 | <0.0001 |
| **Mechanical output** |  |  |  |  |  |  |  |  |
| Interaction | 13.31 | 0.0405 | 39.53 | <0.0001 | 16.60 | 0.0002 | 58.84 | <0.0001 |
| Concentration | 18.58 | <0.0001 | 24.35 | <0.0001 | 29.92 | <0.0001 | 3.505 | <0.0001 |
| Day | 10.33 | 0.0009 | 19.41 | <0.0001 | 15.02 | <0.0001 | 24.30 | <0.0001 |
| **BNP** |  |  |  |  |  |  |  |  |
| Interaction | 13.83 | <0.0001 | 34.04 | <0.0001 | 35.02 | <0.0001 | 29.64 | <0.0001 |
| Concentration | 10.40 | <0.0001 | 63.44 | <0.0001 | 16.22 | <0.0001 | 58.17 | <0.0001 |
| Day | 71.89 | <0.0001 | 0.5562 | <0.0001 | 38.73 | <0.0001 | 3.619 | <0.0001 |
| **NT-proBNP** |  |  |  |  |  |  |  |  |
| Interaction | 17.44 | <0.0001 | 28.93 | <0.0001 | 38.24 | <0.0001 | 24.05 | <0.0001 |
| Concentration | 6.097 | <0.0001 | 64.51 | <0.0001 | 13.78 | <0.0001 | 52.86 | <0.0001 |
| Day | 64.50 | <0.0001 | 3.995 | <0.0001 | 13.77 | <0.0001 | 14.28 | <0.0001 |
| **Cardiac troponin I** |  |  |  |  |  |  |  |  |
| Interaction | 10.73 | 0.3797 | 12.86 | 0.0536 | 8.460 | 0.2000 | 24.42 | <0.0001 |
| Concentration | 3.786 | 0.3374 | 17.90 | <0.0001 | 15.32 | <0.0001 | 25.12 | <0.0001 |
| Day | 3.207 | 0.2790 | 10.61 | 0.0008 | 24.23 | <0.0001 | 21.22 | <0.0001 |
| **FABP3** |  |  |  |  |  |  |  |  |
| Interaction | 34.05 | <0.0001 | 16.14 | <0.0001 | 8.209 | 0.0666 | 14.44 | <0.0001 |
| Concentration | 30.37 | <0.0001 | 75.38 | <0.0001 | 42.21 | <0.0001 | 75.64 | <0.0001 |
| Day | 21.32 | <0.0001 | 6.535 | <0.0001 | 10.58 | <0.0001 | 1.455 | 0.0012 |
| **Basal respiration** |  |  |  |  |  |  |  |  |
| Interaction | 18.82 | <0.0001 | 14.62 | <0.0001 | 16.10 | 0.0056 | 14.22 | <0.0001 |
| Concentration | 0.9849 | 0.5480 | 28.34 | <0.0001 | 1.933 | 0.4591 | 34.68 | <0.0001 |
| Day | 68.53 | <0.0001 | 53.28 | <0.0001 | 62.38 | <0.0001 | 45.78 | <0.0001 |
| **ATP-linked respiration** |  |  |  |  |  |  |  |  |
| Interaction | 20.00 | <0.0001 | 14.66 | <0.0001 | 17.15 | 0.0072 | 20.08 | <0.0001 |
| Concentration | 1.051 | 0.5082 | 30.91 | <0.0001 | 1.733 | 0.5739 | 35.80 | <0.0001 |
| Day | 67.36 | <0.0001 | 50.97 | <0.0001 | 59.59 | <0.0001 | 37.38 | <0.0001 |
| **Maximal respiration** |  |  |  |  |  |  |  |  |
| Interaction | 8.110 | 0.0048 | 20.16 | <0.0001 | 6.819 | 0.5599 | 18.64 | <0.0001 |
| Concentration | 2.618 | 0.0368 | 31.61 | <0.0001 | 9.448 | 0.0058 | 40.51 | <0.0001 |
| Day | 79.61 | <0.0001 | 42.74 | <0.0001 | 59.73 | <0.0001 | 38.54 | <0.0001 |
| **Reserve capacity** |  |  |  |  |  |  |  |  |
| Interaction | 6.511 | 0.1249 | 22.43 | <0.0001 | 10.66 | 0.4001 | 21.43 | <0.0001 |
| Concentration | 6.371 | 0.0018 | 32.78 | <0.0001 | 15.60 | 0.0014 | 41.39 | <0.0001 |
| Day | 73.68 | <0.0001 | 39.38 | <0.0001 | 42.10 | <0.0001 | 34.66 | <0.0001 |

ANOVA, analysis of variance; ATP, adenosine triphosphate; BNP, brain natriuretic peptide; CTG, CellTiter-Glo^®^; FABP3, fatty acid–binding protein 3; LDH, lactate dehydrogenase; NT-proBNP, N-terminal pro–brain natriuretic peptide.

**Supplementary Figure 1.** Seahorse oxygen consumption rate (OCR) average time course traces.
